# Hantavirus Prevalence in the IX Region of Chile

**DOI:** 10.3201/eid0907.020587

**Published:** 2003-07

**Authors:** Marlis Täger Frey, Pablo C. Vial, Constanza H. Castillo, Paula M. Godoy, Brian Hjelle, Marcela G. Ferrés

**Affiliations:** * Universidad Católica de Chile, Santiago, Chile; † Universidad Austral de Chile, Valdivia, Chile; ‡ Universidad del Desarrollo, Santiago, Chile; § Universidad de La Frontera, Temuco, Chile; ¶ University of New Mexico School of Medicine, Albuquerque, New Mexico, USA

**Keywords:** hantavirus seroprevalence, hantavirus asymptomatic infection, hantavirus epidemiology, hantavirus diagnosis, research

## Abstract

An epidemiologic and seroprevalence survey was conducted (n=830) to assess proportion of persons exposed to hantavirus in IX Region Chile, which accounts for 25% of reported cases of hantavirus cardiopulmonary syndrome. This region has three geographic areas with different disease incidences and a high proportion of aboriginals. Serum samples were tested for immunoglobulin (Ig) G antibodies by enzyme-linked immunosorbent assay against Sin Nombre virus N antigen by strip immunoblot assay against Sin Nombre, Puumala, Río Mamoré, and Seoul N antigens. Samples from six patients were positive for IgG antibodies reactive with Andes virus; all patients lived in the Andes Mountains. Foresting was also associated with seropositivity; but not sex, age, race, rodent exposure, or farming activities. Exposure to hantavirus varies in different communities of IX Region. Absence of history of pneumonia or hospital admission in persons with specific IgG antibodies suggests that infection is clinically inapparent.

Hantaviruses, RNA-containing viruses, compose a genus within the family *Bunyaviridae*. The natural reservoirs of the pathogenic New World hantaviruses are rodents of the family *Muridae*, subfamily *Sigmodontinae*, in which a chronic and asymptomatic infection develops (*1*). Hantavirus is a zoonosis transmitted from rodents to humans by inhaling contaminated aerosols from feces, urine, and saliva of infected mice (*2*).

Human infection with hantaviruses have been associated with two diseases. One is the hemorrhagic fever with a renal syndrome (HFRS) caused by Hantaan, Puumala, Seoul, and Dobrava/Belgrade viruses, first recognized during the Korean War in 1950 to 1953. HFRS occurs mainly in Asia and Europe; death rates range from 0.1% to 15% (*3*,*4*). The other disease, a severe respiratory illness known as hantavirus cardiopulmonary syndrome (HCPS), occurs in the Americas and has a death rate of 40% (*1*,*5*–*7*). We prefer the term hantavirus cardiopulmonary syndrome to an alternative term, hantavirus pulmonary syndrome, because most deaths associated with HCPS are related to cardiac failure rather than pulmonary failure, and this aspect of the syndrome remains underappreciated by practitioners and others.

HCPS has been identified in several countries in North and South America and is caused by different hantaviruses: Sin Nombre in North America, Juquitiba virus in Brazil (*8*), Laguna Negra virus in Paraguay and Bolivia (*9*,*10*), Andes in Argentina and Chile (*11*,*12*), Choclo virus in Panama (*13*), and several subspecies or viral genotypes of Andes virus in Argentina (e.g., Oran, Lechiguanas, et al.) (*14*–*16*).

In 1995, Andes virus was first identified in the Argentinean Patagonia and was recognized in central and southern regions of Chile. The reservoir is the long-tailed pygmy rice rat (*Oligoryzomys longicaudatus*), a species that occurs primarily in temperate forest (*17*).

Since HCPS emerged in Chile, 287 cases have been confirmed as of March 2003, causing a substantial impact on the public health system; the death rate for HCPS in Chile has exceeded 40%. In addition to the 287 cases of HCPS, 17 cases of mild infection with no cardiopulmonary involvement have been reported, demonstrating that hantavirus infection may have variability in its expression. Serologic studies have established both clinically asymptomatic infections and symptomatic infections not recognized at the time as hantavirus infection (*18*–*23*).

The phenomenon of clinically nonapparent infections varies in different areas and populations of the Americas. In the United States, the proportion of infection versus disease is thought to be close to 1% (i.e., disease develops in most of the infected patients, and human population seroprevalence varies between 0.2% to 1.7%) (*4*,*24*,*25*). Seroprevalence studies in South America, however, have shown that some populations have had much more frequent exposure to hantaviruses in the absence of known clinical manifestations, as seen in some populations native to Paraguay and Northern Argentina (40% and 17%, respectively) (*26*), the general population in Aysen, Southern Chile (2.0% and 13.1% in urban and rural areas, respectively) (*27*), São Paulo and Bahia, Brazil, (1.23% and 13.1%, respectively) (*28*,*29*), and recently in Panama (30%) (13). The populations with the highest seroprevalence were indigenous persons rather than those of European ancestry.

Two hypotheses have been proposed to explain these phenomena. The first one presumes that strains in South America are of lesser pathogenicity, though in many cases nonpathogenic viruses have yet to be detected in local rodent populations. The second hypothesis involves the existence of at least two variables: the nature of the exposure and genetic constitution of the host population. The high seroprevalence seen in Paraguay (*26*) could be caused by a higher resistance in the aboriginal population and greater exposure to the virus in some regions. The prevalence and high case-fatality ratios seen in North America might be the result of a lower exposure, a lesser genetic resistance to disease, or both.

More than 20 strains of hantaviruses are known worldwide; not all of them are associated with human disease. The observations discussed in this article suggest the need to study whether hantaviruses of lesser pathogenicity and other clinical entities until now unrecognized may occur in areas where the seroprevalence is high and disease incidence is low (*11*,*14*–*16*,*18*). For example, hantaviruses previously thought to be endemic only in Europe and Asia have also been recognized in the North American continent (*30*–*32*).

To study the seroprevalence of hantavirus in Chile, we studied the general population in the IX Region for the reasons that follow. This area is ranked third among those affected by HCPS, accounting for 25% of the known cases in Chile. Three different areas can be distinguished on the basis of geographic features in relation to the two mountain ranges that traverse the region (the Pacific Coast and the coastal range [Coastal], the Central Valley [Central], and the pre-Andean Region [Andean]). The Mapuche, the main native ethnic population of Chile, account for 26.3% of the total regional population. Members of the tribe can readily be identified from surnames, which are derived from both parents. Therefore, we were able to study the seroprevalence of hantavirus in the same region but in areas having different incidence rates of the disease and evaluate the asymptomatic infection in the Mapuche aboriginal population.

## Methods

### Study Design and Area

We conducted a cross-sectional epidemiologic and serologic survey to determine the prevalence of IgG antibodies against hantavirus N antigen in the adults living in nine communities of the IX Region of Chile. The IX Region of Chile is located in the southern part of the country, between parallel 37° and 40° South and the meridians 70° and 74° East (Figure). The region has a surface area of 31,842.3 km^2^ and 781,242 inhabitants in 31 communities; of the total population, 38.71% is rural. The characteristics of these populations can be distinguished according to geographic features. The Mountainside Andes range (Andean) is rural area with small towns; the population there is primarily sustained with farming, woodcutting, and tourist services. The population composes 13.8% of the region’s total population, yet accounts for 82% of HCPS cases. The Central Valley intermediate depression (Central) is a predominantly urban area in which the main sources of income are industry, farming, and ranching. Temuco, the capital and main urban center, is located in this area. The Central Valley contains 67.3% of the total population and accounts for 5.9% of HCPS cases. Coastal inhabitants account for 19% of the region’s population; the community sustains itself with farming and forestry activities, fishing, and tourism. Twelve percent of HCPS cases originate in this area.

**Figure:**
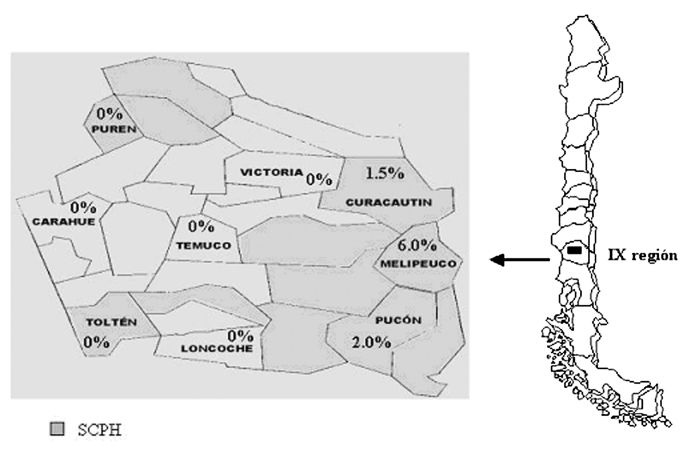
Geographic IX Region, Chile, and hantavirus seroprevalence in the tested communities of the IX Region. SCPH, states for; HCPS, Hantavirus Cardiopulmonary Syndrome.

### Sample Design

The sample included 847 persons, enough to give us accurate point seroprevalence given estimated seroprevalence rates of 7%, 3%, and 5% for the Andean, Central, and Coastal areas, respectively, as derived from previous studies in the country (*27*) and the distribution of HCPS cases in the region. The sample was designed to be representative of each community according to information available from the National Statistics Agency about population sex, ethnicity, age distribution, and rural-urban proportion. Persons were contacted in their homes with a predetermined plan that included the selection of the housing blocks to be studied, the home at which to start enrollment, and how to proceed thereafter. Only one person was enrolled in each household contacted. That person was informed of the study, signed an informed consent, and agreed to answer a questionnaire and donate a sample of blood. Only persons >15 years of age who were free of any current febrile respiratory illness were asked to enroll. The epidemiologic questionnaire, previously validated in the community, requested information about sex, age, race, residency, labor activity, rodent exposure at home or work, cardiorespiratory disease history, hospital admissions, and direct contact with HCPS patients.

Nine communities were included, three per geographic area. Curacautín, Melipeuco, and Pucón (Figure) were chosen as representative of the Andean area (n=277); Victoria, Temuco, and Loncoche from the Central area (n=279); and Purén, Carahue, and Toltén from the Coastal area (n=291). The Andean and Coastal areas were considered rural; the Central area was considered to be a composite of rural and urban. Persons were classified as Mapuches when they had at least one surname of Mapuche derivation. The study was approved by the Catholic University Ethics Committee.

### Collection, Processing, and Analysis of Samples

Blood samples were collected, transported, and centrifuged the same day. Serum samples were kept frozen at –20°C and sent for analysis to the Laboratory of Virology at the Pontificia Universidad Católica de Chile, Santiago, Chile. An enzyme-linked immunosorbent assay (ELISA) was performed to detect hantavirus-specific immunoglobulin (Ig) G antibodies in all samples.

ELISA was performed as described (*33*,*34*) by using recombinant antigen for the Sin Nombre strain (produced in *Escherichia coli* and provided by Centers for Disease Control and Prevention [CDC]), which crossreacts with all American hantaviruses. Briefly, serial dilutions (1:100, 1:400, 1:1,600, and 1:6,400) of patient serum samples were incubated for 1 h at 37°C in antigen-coated, 96-well plates. Peroxidase-coupled anti-human IgG was used as secondary antibody and incubated 1 h at 37°C. After substrate reaction, plates were read at 414 nm.

The net absorbance values are results of the hemiplates absorbance subtraction with and without antigen. To be considered positive, the net absorbance values for a sample had to be >0.2 in the 1:100 and 1:400 dilutions, and the sum of all net absorbance values had to be >0.95. Positive serum samples were tested twice and verified to be positive at CDC.

A confirmatory strip immunoblot assay (SIA) was performed an all positive samples (B.H.). Four recombinant N antigens from Sin Nombre, Puumala, Rio Mamoré, and Seoul hantaviruses were fixed onto a nitrocellulose membrane by vacuum. Serum samples (1:200) were incubated with the strips and alkaline phosphatase-coupled anti-human IgG added. Reactivity was estimated visually, and each band given an intensity value on a four-point scale, as previously described (*35*,*36*). We regarded a sample as positive for antibodies when reactive to both Sin Nombre and Rio Mamoré on the basis of the presumption that antibodies to Andes virus will always react to closely related Rio Mamoré and generally will crossreact with the other sigmodontine rodent-borne virus, Sin Nombre. A sample was regarded as confirmed seropositive when the serum was reactive in the ELISA and confirmed by the SIA. Fisher exact test was used for the analysis of independent variables. A p value >0.05 was considered significant.

## Results

### Sample Population Characteristics

A total of 830 persons were included in the study, with 271, 272, and 286 from the Andean, Central, and Coastal, respectively. The age range was 15–88 years of age (mean 39.4 years of age); 47.6% were men, and 18.3% were of Mapuche origin. In all, 73.3% of the sampled population was considered rural. Men worked in farming, forestry, or both in 61.8% of the cases; 62.2% of the women were housewives. A history of exposure to rodents, their excreta, or both at home was reported in 88.0% of enrolled persons; 54.6% reported exposure at work.

### Serology

Six (0.72%) samples were hantavirus antibody-positive by both ELISA and SIA. The samples came from persons who lived in the Andean area, giving a seroprevalence of 2.15% (6/271) for this area, significantly higher than the other two regions studied (p=0.0001). The seropositive cases belonged to each of the three counties studied; however, relative frequencies varied: Curacautín, 2.6% (2/132); Melipeuco, 6.1% (2/33); and Pucón, 2.0% (2/100). All three counties have previously had HCPS cases reported (Figure). As shown in Table 1, four case-patients were male, and two were Mapuche. The case-patients ranged from 28 to 76 years of age, with a mean of 52.8 years of age. Seropositivity could not be associated with sex (p*=*0.43), race (p=0.30), or age (p=0.18). Five of the case-patients worked in farming or forestry, although only forestry had a significant association with seropositivity (p=0.018), meaning the risk for infection was 10 times higher (relative risk 9.72; confidence interval 1.15 to 82.44). Five of the case-patients said they had been exposed to rodents or their excreta either at home or work. This exposure, however, did not reach statistical significance (p=0.1 and p=1.0, respectively). The sixth case-patient was not exposed to rodents; however, he had been working in a large shed, the woods, and a sawmill; he also had been weeding. Finally, none of the six antibody-positive persons had previous contact with HCPS patients, history of pneumonia, or hospital admission.

**Table 1:** Relationship between independent variables and seropositivity to hantaviruses, IX Region, Chile

Variables	Seropositive (total)	p value	RR (95% CI)^a^
**Sex**			
Male	4 (395)	0.43	
Female	2 (435)		
**Age**			
15–44 y of age	2 (550)	0.18	
>44 y of age	4 (280)		
**Race**			
Mapuche	2 (151)	0.30	
Other	4 (679)		
**Area**			
Andean	6 (271)	0.0001^a^	NA^b^
Central	0 (273)		
Coastal	0 (286)		
**Risk activity or labor**			
Agrarian^c^			
No activity	1 (271)		
Two or more	4 (341)	0.38	
Foresting^d^			
No activity	1 (279)		
With activity	5 (135)	0.018	9.72 (1.15 to 82.44)
Exposure to rodents			
Peridomestic	5 (731)	0.10	
Laboral	5 (453)	1.00	

^a^p <0.05; p values were determined by using 2-tailed Fisher exact test; RR, relative risk; CI, confidence interval. ^b^Not applicable. ^c^Clearing, shrubbery cutting, working in pastures or cellars. ^d^Foresting, working at a sawmill.

## Discussion

This study shows serologic evidence of past infection by hantavirus in the Chilean population. The hantavirus associated with symptomatic infections in Chile is Andes virus, identified in patient blood samples by reverse transcription-polymerase chain reaction (RT-PCR) and recently isolated from human blood (*12*,*37*).

Our results show the global seroprevalence to hantavirus in the adult general population in the IX Region to be 0.72% (ranging from 0 to 6%, depending on the community). Seroprevalence is significantly higher in the Andes rural area, consistent with the observed elevated incidence of HCPS disease in this area of Region IX between 1997 and 2000 (Table 2). This geographic distribution is probably related to the great tracts of native forest, where *Chusquea quila*, a bamboo-like shrub that protects and feeds the carrier rodent, is abundant. Moreover, the increasing development of the forestry industry plus an increase in the rodent population caused by the favorable climatic condition because of the El Niño effect have caused humans and mice to interact closely.

**Table 2:** Incidence of hantavirus cardiopulmonary syndrome (HCPS) and seroprevalence in Chile’s IX Region according to geographic area

	Region				p value^a^ (A vs. B)	RR (95% CI)^a^
	Global	Andean (A)	Central (B)^b^	Coastal		
**Disease**					0.0001	29.6 (11.46 to 76.47)
Cases	34	29	1	4		
Population	781,242	127,974	424,278	229,190		
Cumulative Incidence (1997-2001) (x10^6^)^c^	4.35	22.66	0.23	1.74		
**Infection**					0.00013	NA^d^
Seropositive	6	6	0	0		
Sample	830	271	272	286		
Prevalence (%)	0.72	2.15	0	0		

^a^p values were determined using 2-tailed Fisher exact test; RR, relative risk; CI, confidence interval. ^b^B is Central and Coastal regions combined. ^c^Chile’s Department of Health, March 2001. ^d^Not applicable.

We did not find an association between seroprevalence and reported exposure to rodents, probably because this type of exposure is frequent in all groups in the region, occurring both at work and at home. The exposure to rodents or their excreta is necessary but apparently not sufficient to acquire the infection. Presumably an exposure to a specific reservoir mouse or virus carrier, human behavior, and biologic factors are important.

Forestry is associated with a higher risk for infection. This labor, whether in the woods or at sawmills, is performed almost exclusively by men; both men and women share farm work in most instances. This finding may explain why a higher proportion of HCPS develops in men (75% of reported cases). However, any apparent association between hantavirus exposure and a particular occupation does not necessarily implicate these occupations as a risk factor. In fact, occupation may be a marker for living, sleeping, or housing conditions that constitute the proximate risk factor for exposure. Indoor exposure to rodents is common in patients with HCPS (*38*).

On the basis of these results, we argue that infections by hantavirus follow a gradient of exposure to the virus in the IX Region. Our population has a different epidemiologic profile to those of the aborigines of Paraguay (40%) and North Argentina (17%) in South America, which have low seroprevalence, similar to that described for North American populations, as reported by Vincent (*13*) and Ferrer (*26*). This lower seroprevalence could be because of a greater pathogenicity and clinical severity in infections by the prototypical (southern) form of Andes virus, similar to Sin Nombre virus.

Less pathogenic hantaviruses may cause a greater amount of asymptomatic infections, as seen for HFRS in Europe and Asia (seroprevalence 7.9% to 10%, death rate, 0.1% to 15%) (*39*) and some American hantaviruses (Laguna Negra in Bolivia and Paraguay, Choclo in Panama, Oran and Lechiguanas in Argentina) for which seroprevalence is high and case-fatality ratios are <30% (*9*,*13*,*15*). This finding is in contrast to the findings of hantaviruses with severe clinical syndromes and high death rate (i.e., Sin Nombre and Andes viruses, both of which have been associated with few subclinical infections). The lack of subclinical infections can be caused by a variation in virulence or by different genotypes of the hosts, which give them greater resistance to infection and disease.

A slightly disproportionate fraction of seropositive samples (33%) were from Mapuche (18.3% of those sampled were Mapuche). Moreover, when both hantavirus seroprevalence and the HCPS incidence rates in this region are considered, more infection but less disease (not significant) is found in the Mapuche population (Table 3). Future epidemiologic studies should address this finding and use a larger sample to evaluate possible associations between racial origin and the incidence rates of infection and disease.

**Table 3:** Incidence of hantavirus cardiopulmonary syndrome (HCPS) and seroprevalence in Chile’s IX Region according to race

Race	Seroprevalence	Incidence	HCPS cases/population	RR (95% CI)^a^
	Positive/total (%)	RR (95% CI)^a^	(rate x 100.000)	
Mapuche	2/151 (1.3)	2.23 (0.41 to 12.06)	4/205,466 (1.9)	0.37 (0.13 to 1.06)
Other	4/678 (0.6)	–	30/575,776 (5.2)	

^a^Relative risk (95% confidence interval). p values were determined by using two-tailed Fisher exact test; RR, relative risk; CI, confidence interval.
